# Editorial: Endocrine organoids for modeling, drug development, and treatment of cancer and other diseases

**DOI:** 10.3389/fendo.2023.1292619

**Published:** 2023-09-25

**Authors:** Vijaya Kumar Pidugu, Anil Mathew Tharappel, Sonam Kumari, Kumar Sanjiv

**Affiliations:** ^1^ Laboratory of Cancer Biology and Genetics, Center for Cancer Research, National Cancer Institute, Bethesda, MD, United States; ^2^ Department of Pharmacology and Toxicology, R. Ken Coit College of Pharmacy, The University of Arizona, Tucson, AZ, United States; ^3^ National Institutes of Health, Bethesda, MD, United States; ^4^ Scilifelab, Department of Oncology, Pathology, Karolinska Institute, Solna, Sweden

**Keywords:** endocrine organoid models, patient-derived organoids, stem cells, pituitary gland, endometrium, papillary thyroid cancer, colorectal cancer, ECM matrix scaffold

The main components of the human endocrine system are comprised of the pituitary, endocrine glands, hypothalamus, and hormones that are critical for the regulation of body metabolic functions by performing a complex network of molecular signaling cascades regulated by hormones released by the endocrine glands (1). The endocrine glands such as adrenal glands, thymus, thyroid glands, pancreas, prostate, ovaries, and testis interact with each other in an extremely coordinated manner. The loss of synergism and inappropriate function of the endocrine system leads to the excessive or deficient production of hormones by the endocrine glands resulting in endocrine disorders such as diabetes, hypothyroidism, hyperthyroidism, polycystic ovary syndrome, acromegaly, menstrual disorders, osteoporosis, and endocrine cancers, etc. The common endocrine cancer types include thyroid cancer, pituitary tumors, adrenal cancer, pancreatic cancer, parathyroid cancer, hypothalamic endocrine tumors, ovarian, and prostate cancer. Although most cases of endocrine tumors are benign, prostate, ovarian, and pancreatic cancers are the leading cause of cancer-related deaths in the US. Both benign and metastatic endocrine cancers cause serious problems in patients by abnormal hormone production and impairing many critical physiological processes such as growth, development, sexual functions, metabolism, and reproduction.

The complexity of the endocrine system is the main limitation for understanding and treating endocrine diseases. Hormone therapy is the long-established treatment option for endocrine disorders with insufficient hormone secretion. However, taking into consideration the high complexity of the endocrine system, hormone injections may not be enough to reconstitute endocrine function, and severe side effects may occur to the patients treated with hormone therapy. Thus, stem cell or organ transplantation has been a widely advised approach for treating endocrine diseases. Hypothyroidism and hypoparathyroidism have been successfully treated by auto-transplantation of stem cells or organs. However, allotransplantation was not successful due to limited organ supply and immune rejection. Therefore, stem cell-derived endocrine organoids have been an advanced approach to overcome the limited organ supply. Organoids are 3D clonal outgrowth structures in which cell clusters originating from parental pluripotent or tissue progenitor stem cells are grown in an *in vitro* culture system with the support of an extracellular matrix (ECM). Endocrine organoids maintain autochtonous tissue structure as they contain stem cells differentiated into multiple cell types with 3D cell arrangements and cell-to-cell interactions to mimic the native organ. Hence, distinct endocrine organoid cultures have been established for modeling multiple endocrine diseases, preclinical drug screening, and cell transplantation therapies. The patient-derived endocrine organoids are widely used as new therapeutic tools for evaluating drug response and the development of personalized medicine. In this regard, the present special Research Topic aims at providing recent developments in the endocrine organoid field. Overall, the research and review articles collected in this Research Topic present insights into the advancement of methodologies for endocrine disease modeling for a better understanding of disease progression, drug development, personalized medicine, and future perspectives for effective treatment strategies.

Hypothyroidism is a well-known endocrine disease characterized by thyroid hormone deficiency. Although the simple daily thyroid hormone injection is a common treatment, several patients undergo harmful side effects due to inaccurate hormone levels in their bloodstream. Therefore, there is an urgent need to develop novel innovative treatment strategies for patients with hypothyroidism. Li et al. presented a systemic review by collecting experimental data from 66 independent research studies related to transplanting engineered thyroid tissues from adult tissues of multiple species, embryonic, newborn, and fetal tissues into recipients. By comparing 2D thyrocyte cultures with 3D organoid cultures, authors have found that thyrocytes in 3D cultures recapitulate the normal physiological thyroid function. This study highlights promising results of current thyroid reconstruction models and transplantation of an engineered thyroid for the recovery of optimal thyroid hormone levels in recipients. In addition, this study also describes the efficacy and potential tumorigenicity of *in vivo* transplantation of thyroid-derived organoids in xenograft mice models to understand thyroid carcinogenesis in early and prolonged cultures of thyroid organoids. This review suggests that prolonged culturing of thyroid organoids does not alter the expression of tumor-related markers.

Papillary thyroid cancer (PTC) is the most common pathological subtype of thyroid cancer accounting for 85% of all reported cases. The surgical removal of thyroid tumors followed by radioiodine therapy is the primary mode of treatment. However, a portion of patients have been reported to develop distant metastasis due to a lack of response to radioiodine treatment. For many years, research studies have been carried out with conventional 2D cell line models that were known to have many drawbacks as they do not represent the genetic and histological characteristics of PTC. Hence, there has been an increased attention and thrust for research studies on patient’s tumor-derived organoids in the past several years. Yang et al. have successfully developed step-by-step methodologies for the establishment of patients-derived PTC organoids from thyroid cancer clinical samples. With the optimized protocol, Yang et al. were able to achieve a success rate of 77.6%. Moreover, PTC organoids have shown histological and mutational status identical to parental PTC tumors.

Colorectal cancer (CRC) is one of the most lethal malignancies with high mortality worldwide. The high microsatellite instability (MSI-H) tumors carry heritable gene mutations affecting proteins (MLH1, MSH2, PMS2, and MSH6) involved in the DNA mismatch repair (MMR). Loss of MMR protein function is termed MMR-deficient (MMRd). Lynch syndrome (LS) patients have a hereditary predisposition to develop cancer due to germline mutations disrupting MMR protein functions. However, multiple clinical studies have shown that MSI-H patients are associated with better response to long-term immunotherapy response and improved prognosis in CRC. Recently, anti-PD-1/PD-L1 inhibitors such as pembrolizumab and nivolumab have been approved by the FDA for the treatment of MSI-H metastatic solid tumors and colorectal cancer patients respectively. Although immune checkpoint blockade (ICB) therapy has boosted the enthusiasm for cancer treatment, only a portion of patients have features of MSI-H, and some ICB-responsive patients are still difficult to distinguish. Therefore, careful analyses of multiple markers to get an ideal strategy to identify patients sensitive to ICB treatment must be performed. To better understand the molecular and resistance mechanisms to ICB treatments of MMRd cancers, Song et al. established organoids and an orthotopic mouse model from intestinal tumors generated in an MSH2-deficient mouse model. The MSH2-deficient organoids have been shown to have typical characteristics of MSI-H cancers such as high frequency of frameshift mutations, genome instability, and tumor heterogeneity. Moreover, the orthotopic cecal implantation of tumors from organoids exhibited profound tumor growth with distant metastasis to lymph nodes and the liver. Overall, this study described that Msh2-deficient organoids and an orthotopic mouse model could be appropriate for preclinical testing of anti-cancer drugs and neoantigen-based vaccines.

The pituitary gland is the core component of the endocrine system, and it regulates multiple physiological functions including metabolism, reproduction, growth, and lactation, etc. Because of the pituitary’s wide range of functions in the endocrine system, malfunctioning of the pituitary gland such as hypopituitarism and hyperpituitarism causes serious problems in the body. Whilst most of the pituitary tumors are benign, the symptomatic pituitary adenomas cause increased mortality and morbidity in patients due to hypo or hypersecretion of hormones. Therefore, pituitary adenomas are treated with surgical removal and radiation therapy. Multiple *in vitro* 2D cell culture formats have been developed to unravel the pathobiology of pituitary tumors. However, cell line models failed to capture the full spectrum of histology as the cell lines represent only one type of pituitary cells. Over the last decade, technological advancements have led to the development of 3D organoid models from multiple cell origins including embryonic stem cells (ESCs), induced pluripotent stem cells (iPSCs), tissue-specific stem cells (TSCs) to study healthy and disease conditions of the pituitary gland. Laporte and Vankelecom reviewed the latest developments in the field of pituitary organoids and their cutting-edge translational applications for understanding pituitary biology, development, and disease progression. Comprehensively, this review summarized the latest pituitary organoid models and discussed the applications of these powerful tools in translational research.

The endometrium is an essential tissue that lines the uterus and plays a critical role in mammalian reproduction that undergoes dynamic changes in the menstrual cycle and embryo developmental stages. Despite its vital role, molecular and endometrial biology in healthy and diseased states is not adequately understood. Recently well-established endometrium-derived organoid models have been generated by embedding stem cells in an ECM scaffold. However, endometrial ECM is composed of different protein components in proliferative and secretory phases. Therefore, the lack of consistency of matrix scaffolds is the main constraint for potential translational applications of endometrium-derived organoid models. Hence, researchers must develop well-defined ECM scaffolds for reliable and optimized growth of endometrium-derived organoids. De Vriendt et al. reviewed the recent updates on both natural and synthetic ECM scaffolds that have been optimized for the faithful and efficient growth of endometrium-derived organoids. This mini review extends our knowledge of advancing precisely defined ECM matrix scaffolds and their applications in basic and clinical research to address research questions of endometrium pathophysiology.

## Conclusion

The research in the field of endocrine organoids has been rapidly advancing in recent years with emerging powerful tools including CRISPR gene editing, single-cell sequencing, iPSC differentiation, synthetic ECM matrix scaffolds, and 3D culture systems. In addition to the production of 3D organoids, new strategies are also being developed for the transplantation of endocrine organoids and tested in preclinical as well as clinical platforms. The endocrine organoids enabled a better understanding of the underlying molecular mechanisms of diseases related to the endocrine system. Endocrine organoids have a lot of potential to upgrade effective therapeutic strategies and advancements in this field can result in promising translational perspectives. For organ transplants, limited availability of organs and complicated surgical procedures are involved. The *in vitro* 3D organoid culture system provides an unlimited supply of endocrine tissue. Therefore, stem cell-derived endocrine organoids have the potential to overcome the limited organ supply in the future.

## Future perspectives

Although the endocrine organoid technology might provide revolutionary models for multiple endocrine diseases, there is an unmet need for the development of models for adrenocortical carcinoma (ACC), medullary thyroid carcinoma (MTC), parathyroid hyperfunctioning, and adrenal neoplasms which can serve as *in vitro* tools for developing targeted therapy strategies. Patient-derived 3D organoids are closer to native organ structures as compared to cell lines. However, there are many hurdles to overcome in the development and use of these endocrine organoids in the clinic. Most endocrine organoid studies are mainly focused on pancreatic organoids for treating diabetes, while the endocrine organoid field is still in its evolving state as treating endocrine disorders has limitations. The immune response is one of the major roadblocks for transplantation models. Lately, encapsulated endocrine organoids have been reported to have reduced immune response. Co-transplantation of immunomodulatory cells along with the endocrine organoids is in the early stages of testing. There is requisite for research studies focusing on testing vascularization to provide oxygen and nutrients by transplanted organoids. The cost of 3D cultures is expensive and lacks well-optimized culture conditions and media compositions. The development of cost-effective synthetic hydrogel scaffolds alternative to natural ECM and advanced high-throughput technologies that require low quantities of ECM scaffold could overcome these limitations. The continued advancements of new technologies in the endocrine organoids field will bridge the gap between bench to bedside in the future.

## Author contributions

VKP: Conceptualization, supervision, and organizing the Research Topic, Writing-Original draft, writing-review & editing. AT: Writing – review & editing. SK: Writing – review & editing. KS: Writing – review & editing.
